# Where Do I Go When My Doctor’s Office Is Closed? The Availability of Out-of-Hours Care Information on Primary Care Practitioners’ Websites

**DOI:** 10.2196/50857

**Published:** 2024-05-29

**Authors:** David Legg, Hendrik Napierala, Felix Holzinger, Anna Slagman

**Affiliations:** 1 Health Services Research in Emergency and Acute Medicine Charité - Universitätsmedizin Berlin Germany; 2 Institute of General Practice and Family Medicine Charité - Universitätsmedizin Berlin Germany

**Keywords:** out of hours, primary care, telephone triage, websites, care information

## Introduction

Despite the relative longevity of Germany’s medical on-call number 116 117, evidence suggests that awareness of this helpline for non–life-threatening emergencies remains limited [1]. In light of this, this study turns to the often overlooked medium of primary care practitioner (PCP) websites to promote this service. While these websites may have previously functioned as little more than “electronic nameplates” [2], as the use of primary care online services, such as online appointment booking, continues to grow, so too does their potential to promote and reinforce beneficial health-seeking behaviors through the provision of information. With this in mind, the aim of this research is to quantify the availability of out-of-hours (OOH) service information currently provided on PCP websites in Berlin, Germany.

## Methods

A comprehensive list of primary care physicians practicing in Berlin and Brandenburg in May 2023 compiled by Charité’s Institute of General Practice and Family Medicine was used as a sampling frame to quantify OOH service information. A simple random sample of 10% of the total list was then generated using the SPSS (version 27; IBM Corp) random sample function. Google searches were then conducted using the doctor’s title, surname, and address to identify practices with websites. Website links were then collated with the preceding practice information into an SPSS database.

The variables of interest were information (including links to further information) concerning opening hours, OOH service information, and mentions of Germany’s nonemergency number. Search terms included “116 117,” “116117,” “Opening Hours,” and “Consultation Hours.” OOH service information was defined as directives concerning what to do when the service is not available.

To quantify the availability of the above, a two-stage approach was used. In the first stage, the Google search site operator was utilized to search the entire website for mention of the medical on-call number. In the second stage, manual close reading of each PCP website’s home page was conducted alongside the use of the find command (Ctrl + F) to identify the variables of interest. Home pages were selected for further analysis as these are often a website’s default loading page and, as such, provide service users with immediate information.

After the primary researcher (DL) coded all the relevant data to ensure intercoder reliability, a second researcher (HN) coded 10% [3] of the valid sample. Any discrepancy was resolved by consensus. Further details of the study’s methodology are available in the study protocol [4].

## Results

**Figure 1:**
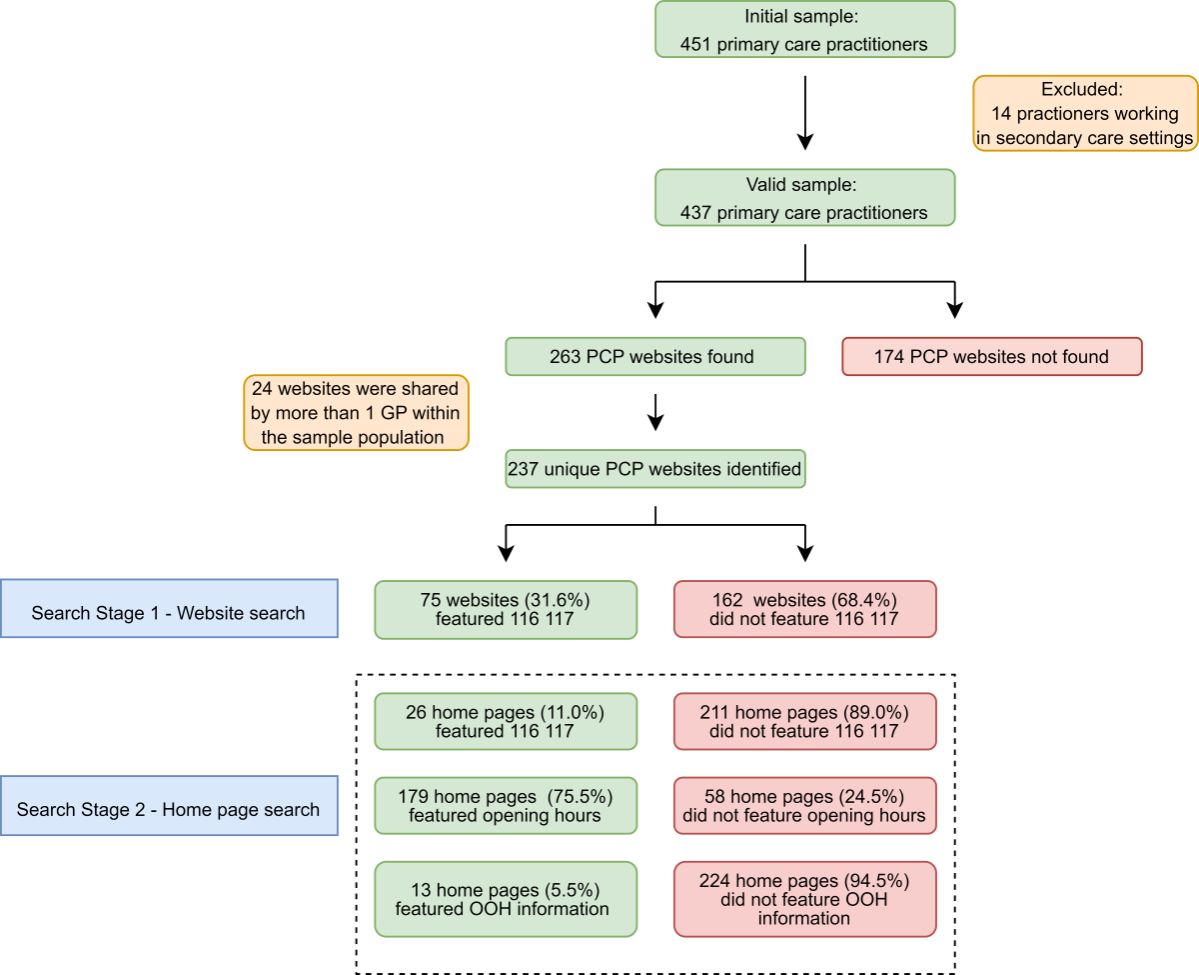
OOH service information featured on primary care practitioners' websites. GP: general practitioner; OOH: out-of-hours; PCP: primary care practitioner.

In total, the sampling frame contained details of 4508 physicians working in Berlin and Brandenburg (Figure 1). After selecting a random sample of 10% [5], 451 PCPs were included for analysis. A further 14 PCPs were excluded from the analysis after they were identified as working in a hospital or inpatient setting (n=437).

Within the valid sample, websites were found for 263 (60.2%) PCPs. Functioning websites could not be found for 174 (39.8%) PCPs. After the removal of duplicates, 237 unique websites were identified: 213 PCPs had their own website and 51 PCPs shared a website with at least one other PCP in the valid sample (n=24 websites).

A search of the entire website for the presence of 116 117 indicated that 75 (31.6%) websites featured references to the nonemergency number; 26 (11%) featured this information on their home page. Information on opening hours was more common: 179 (75.5%) websites featured information or a link to the practice’s opening hours on their home page. Overall, 13 (5.5%) websites provided information about what to do outside of working hours on their home page, and all did so with reference to 116 117, explicitly connecting OOH care with the nonemergency number.

## Discussion

The results of this study indicate that a substantial number of PCPs in Berlin are not providing adequate and accessible OOH service information on their websites. While the ability of these websites to change behavior should not be overstated, PCPs are missing an opportunity to promote and reinforce beneficial health-seeking behaviors by not providing such. The inclusion of OOH service information would create the opportunity for service users to store and recall this information when they perceive the need for medical attention through a medium, which when compared with traditional forms of marketing health care [6], offers a cost-effective means of distributing information that can be updated to reflect changes in service provision as required.

Further research into PCP websites is required. As these results are limited to Berlin, it is unclear whether this information deficit extends nationwide. Future research should also include online services offered, service characteristics, and user estimates.

## Abbreviations

OOH: out-of-hours
PCP: primary care practitioner
